# Translations from Foreign Exchanges

**Published:** 1883-05

**Authors:** 


### Translations from Foreign Exchanges.

BY A. LAGORIO, M. D.

---

### DERMOID CYST OF THE OVARY, OPEN BY SUPPURATION THROUGH THE ABDOMINAL WALL.—CURE.

The following case is reported by Dr. A. Turretta, of Trapani: Mrs. E. L., aged 27, menstruated at 15, had three children with normal pregnancies. During the last pregnancy noticed, the abdomen, greater than previous times, had a conoid form protruding forward. After labor, the abdomen remained more voluminous than ordinary, and since then menstruation ceased. When admitted to the hospital she was pale, amaciated and had an extremely suffering aspect, with dyspnoea. The circumference of the abdomen around the umbilicus was 84 centim.; four inches below, 92 centim. The pubo-umbilical distance, 95 cent.; umbilico-xiphoideal, 16 cent. Skin is distended and red. Palpation is tender and painful in the portion of the abdomen below the umbilicus. The hand reveals an exaggerated heat, with an elastic softness, and a sensation of a superficial abscess. Vagina normal, the neck of the uterus slightly flexed to the right. An incision 4 cent. long was made 8 cent. below the umbilicus, and with an impetus was discharged about six liters of a purulent, viscous liquid of a specific gravity of 1,017, rich in purulent corpuscles and cholesterin crystals. The cavity was washed with carbolized water, and a large drainage tube placed in position. After 27 days, there was some elevation of the temperature, with rigors which were repeated the following day; the secretions changed in color and odor in such a manner as to arouse the suspision of ichorhæmia. The wound and cavity were immediately washed with a solution of pernanganate of potassa, and large doses of quinine administered internally. The rigors ceased, and in a week the temperature became normal, and the patient quickly improved. The uterus remained a little ante-flexed, but no longer painful. The quantity of the discharge diminished considerably, so that the patient, thinking herself cured, begged to leave the hospital after ninety days. A drainage tube was left in the aperture, and the patient advised to continue detergent injections. Three months afterwards, the patient again called at the hospital, saying that for some days she had been tormented with fever, and had with surprise observed the escape from the wound of long hairs with offensive pus. The cavity was examined, and from the aperture escaped a moderate amount of yellow liquid, offensive in odor, composed of pus, fat, etc., with a quantity of long, hard, black hairs, resembling those of the scalp of the patient. This revealed the essential character of a compound cyst; no longer a simple calloid cyst, but combined with another of a dermoid nature. The escape of hairs and liquid continued for about 40 days. Some injections of tincture of iodine were also used. The patient soon gained in strength, and after 70 days the encysted mass constituted only a tumor the size of a lemon, and a fistulous opening. The patient was discharged almost cured. The menstruation reappeared and continued with a perfect regularity.

Dr. Turretta has never noticed in surgical literature such a typical case, of a cure of a compound cyst, by spontaneous suppuration, opened through the abdominal walls, as the one he has reported. Five years have now elapsed since her discharge from the hospital, and he has found the same condition of the mural residual tumor, of the same volume, and the same small fistulous sinus which the patient keeps open with great care with a small elastic tube, fearing that total closure may bring back the old disease.

It is evident that such a fortunate result was due to the suppurative inflammation developed in the tumor. Twisting of the pedicle is sometimes a cause of inflammation of the cyst. But the torsion may be partial so as to produce only an incomplete circulatory strangulation; or may be complete as to produce a complete strangulation, intercepting the arterial circulation, producing statis in the venous capillaries, and therefore hæmorrhage, thrombosis, gangrene of the cystic walls and ichorrhæmia. Traumatic causes are the most common for inflammation of the ovarian cyst. This patient denied having received any blow or wound in the abdomen; but being a farmer was obliged several times to lift and carry around the body heavy objects, which, according to the author's mind, constituted a mechanical stimulus capable, especially if repeated and associated with atmospheric bad surroundings, of producing an inflammatory action. The sudden and strong contracting of the abdominal muscles constituted by themselves a trauma, which sometimes has produced a rupture of the cyst. The peritonitis must have been very limited. The general phenomena were due to the phlegmonous inflammation and to the absorption of the purulent contents.

The fortunate spontaneous cures of the ovarian cysts, although rare, are not entirely new. Broca, referring to statistics of Cherean, says that of twelve cases of cysts, ruptured in the intestine, in the vagina, or through the abdominal walls, five were cured, five returned, and in two death resulted (Traité de tumeurs, Paris, 1869, vol. 11, p. 147). But it seems that they were simply serous cysts, as Broca, at p. 146, op. cit., speaking of the possible accidents of cysts, and of adhesions of the internal parietes, says: "*Terminaison fort rare qui n'est possible que dans les kystes séreux.*" And on page 147 speaking of the curative treatment of the dermoid cysts, adds: "*La course de ces kystes étant très réfractaire á l'inflammation adhésive, ét la récidive étant imminente toutes les fois qu'une partie même très limités de cette parois reste en place au fond du kyste,*" etc.

Koeberlé, Clay, Baker-Brown, Perruzzi, Spencer-Wells, etc., are unanimous in the opinion that only unilocular cysts with serous contents may, in rare cases, recover without ovariotomy; and that the multilocular, the colloid, the compound and the dermoid have no other resource but the radical operation.—*Giornale Internazionale delle Scienze Mediche.*

### The Treatment of Typhoid Fever.

M. Jaccoud explains his treatment of typhoid fever used during the past sixteen years. Two essential characters are present in typhoid fever: The *adynamia*, produced by the typhoid infection, and the febrile consumption; *calorification*, which interferes in the functions of the heart and the brain. From this, two indications are given, one to sustain the strength of the organism, the other consists in the necessity of combating the fever. For the adynamia he advises wine, bouillon and milk; alcoholics (30 to 80 grammes a day), and 3 to 4 grains of extract of quinquina in the 24 hours. For the fever heat he advises 4, 6, 8 or 10 lotions a day of aromatic vinegar. The effect is certain. If the symptoms persist and become worse, he uses quinine in the form of bromhydrate and salicylic acid. He gives the quinine for two or three consecutive days. The first day he gives 1 gram, ½ to 2 grams the second, and third day diminishes the dose to 50 centigrams. The dose is given within 30 minutes; in the morning to obtain the effect in the evening; or in evening to have the effect in the morning. Prefers the salicylic acid if the patient shows no alcoholism, nor weakness of the heart, nor any trouble within the kidneys or brain. If complications about the lungs appear, he applies dry cups on the stomach, and on the superior members.

He has treated in 16 years 655 cases of typhoid fever, and has lost 71 patients; 11 per cent.

To show his excellent results, and to compare it with that of others, he has collected 80,000 cases, and has found that the mortality is 19 to 20 per cent.

He protests against the excessive therapeutical methods that have been used during later years.—*Lyon Médical.*

---

### Past Partum Hæmorrhage Treated with Hypodermic Injections of Ergotinine.

At a meeting of the Académie de Médecine, Dr. Charbazian read a paper on the subject. Ergotinine is the alkaloid of ergot; it is insoluble in water, but soluble in alcohol and chloroform.

A pound of powder of ergot contains 18 centigrams of ergotinine. The indication for its use is when the post partum hæmorrhage is due to the contraction of the uterus. The dose for a single hypodermic injection is 5 to 6 drops of a solution containing 4 milligrams of ergotinine to each twenty drops of the liquid. The injection can be repeated if necessary, but never more than twenty drops. More energetic and permanent contraction of the uterus is produced by it, and its action is more rapid than with the ergotin (which is nothing but an extract of ergot), and it neither produces abscess nor induration. Ergotinine is to ergotin what morphine is to the thebaic extract. The uterine contraction appears within two to five minutes. Could not say how long they last.—*Annales de Gynécologie.*

---

WEISS—THE MICROBIUM OF BLENNORRHAGIC PUS.

The presence of microbes in blennorrhagic pus has been indicated several times. A recent work of the author confirms the studies already made on this subject. The examined pus was taken from men and women with all the necessary precautions. In all cases the microscope revealed, between the pus globules and the epithelial elements, little bodies, some isolated, some united two by two, or forming little groups, and disposed in a special manner, and always with a characteristic aspect. He has examined the pus of thirty-two patients, and every time has discovered these parasitic forms. As a comparison, he has examined the pus of a single metritis, balanoposthitis, chancre or chancroid, leucorrhæa, etc., etc., and has never found the special element, which he considers as characteristic of blennorrhagia. For treatment, he insists upon the parasitic action of the permanganate of potassium. In fact, in all cases of vaginal blennorrhagia treated, in the injection of a solution of 25 centigr. per 1,000, he has noticed a rigid and notable diminution of the microbes; at the same time they lost their zone of involucrum, and were modified so as to indicate their alteration, or their destruction, by the application of the substance.—*Ann. de Dermat.*

### QUASSINE AND ITS USES.

Quassine is the active principle of *quassia amara.* It is amorphous or crystallized. Both forms produce the same effects; the former is preferable at a dose of four to ten cent. gr. a day; of the latter a dose above two cent. gr. produces toxic effects. In a healthy man quassine produces during the first days a rapid increase of the appetite, a more complete digestion of aliments and a rapid development of strength. At a dose of four cent. gr. before meals, it increases the alvine discharges, and therefore becomes useful in constipation caused by a feebleness of the muscular tunic of the intestines. This property is a precious one, for it permits, in many cases, to substitute the quassine for purgatives, which frequently render the constipation invincible, without speaking of the returns which most often are produced after their administration. At the same dose of four cent. before meals, quassine has been given to patients having three or four diarrhœal discharges within twenty-four hours. After eight days of treatment the discharge became normal. Other experiments have proven that quassine has a most pronounced diuretic effect; that it increases the secretion of the salivary glands, of the fauces, of the kidneys, and also of the mammary glands. Quassine is a bitter tonic, aperient and stomachic. It must not be administered during the acute stages of diseases, but in the general debility, the atonic dyspepsia, the anorexia, the chlorosis, the spasmodic vomiting, the long and difficult convalescence, especially of fevers. -*Gazette des Hôpitaux.*

---

### OXIDE OF ZINC POISONING.

At a meeting of the *Société de Médecine de Nancy*, Dr. Spillman communicated his observation of a patient who presented toxic symptoms after the ingestion of a certain quantity of powdered oxide of zinc. N., aged 23 years, was affected with a syphilitic chancre. He was ordered Ricord's pills and an application to the sore of powdered oxide of zinc. The patient, a German, not understanding the directions, when at home drank about ten grains of the powdered oxide of zinc in a glass full of water. After a few moments he was taken with severe pain across the stomach, fainted and fell into a collapse. The druggist who furnished the powder gave to the patient whites of eggs. The doctor saw the patient several hours after the accident; he had cold extremities, pulse small, and continued vomiting. The vomiting persisted for over forty-eight hours, in spite of the means employed (ice, opium, etc.). It was evident that the powdered oxide of zinc had been transformed into a chloride of zinc. An adult secretes about 13,000 to 15,000 gram. of gastric juice daily, which contains four to five grams of hydrochloric acid per 1,000. The albumen, taken in time and in large quantity, had transformed the zinc salt into an insoluble albuminate.—*Revue Médicale de l'est.*

LARYNGEAL SPASM IN A CASE OF LOCOMOTOR ATAXY.

The patient, a mariner fifty-seven years of age, contracted syphilis eight years ago. He worked a good deal in cold weather, and suffered from pains in the lower extremities. Gradually the pains increased to a certain intensity, but they disappeared after two months' treatment. Three years ago, he suddenly had laryngeal spasms, with symptoms of locomotor ataxy. These spasms became frequent, and they were followed by intense pains in the limbs. There was now paralysis of the motors of the eye, with dilatation of the pupils and diplopia of the right side. The troubles in the lower limbs were doubtless of a tabetic character. There was spasmodic constriction of the larynx, but he retains consciousness. Sometimes speaking will produce these attacks, and the inferior vocal cords were influenced. There was vomiting, but no gastric pains.—*Lyon Médicale.*

SOME POINTS IN GENERATION.

Pflueger, the well-known physiologist, has experimented with the eggs and the sperm of the frog. The eggs were obtained from four different places, and also the sperm. Pflueger finds that neither climate, or water, or nutrition, is of any importance as respects the question of sex. He further calls the attention of physiologists to the fact that there is hermaphroditism in the frog until the second year, and that this hermaphroditism is more or less prolonged in different localities. He says, further, that diluted semen acts nearly as well as the concentrated, and that the sex in the frog is generally predetermined.—*St. Petersburg Med. Wochenschrift.*

DIPHTHERIA TREATED BY HOMŒOPATHS.

By request of the homœopathic physicians at St. Petersburg, the Russian government gave these gentlemen a fair trial in all the hospitals of that city. They promised to prove that their treatment of diphtheria was far more successful than that of regular physicians. The commission nominated to supervise the results consisted of government physicians, and in large part of other government officials, who were independent of any outside influence. The result was disastrous for the homœopathic physicians, three-fourths of the children having died under their treatment. The commission, therefore, was forced to declare that after two months' trial, they have found the homœopathic teatment of diphtheria to be a "dangerous fraud."—*Wradshebrija Wedomosti.*

---

IN the monthly report for March of the Bureau of Vital Statistics, we notice the following items:

Total number deaths, 968. Children under five years of age, 483. Greatest number of deaths from phthisis, 94; diphtheria, 49; scarlet fever, 35; pneumonia, 70; infantile convulsions, 86; infantile marasmus, 13; bronchitis, 52; typhoid fever, 20; inanition, 35; by violence, 49; small pox, 9; 1.727 deaths in 1,000. Deaths in March, 1881, 876; March, 1882, 1271; February, 1883, 859.

---

CHIARI, the pathologist, who has just been made professor at Prague, is a man thirty years old who has already made over 8000 post-mortem examinations. At a recent supper given in his honor, one of the speakers said he could not wish for greater happiness than that of being post-mortemed—if one may use the expression—by his friend Chiari.—*Medical and Surgical Reporter.*

